# Differential Fiscal Performances of Plausible Disaster Events: A Storyline Approach for the Caribbean and Central American Governments under CCRIF

**DOI:** 10.1007/s41885-023-00126-0

**Published:** 2023-05-11

**Authors:** Stefan Hochrainer-Stigler, Qinhan Zhu, Alessio Ciullo, Jonas Peisker, Bart Van den Hurk

**Affiliations:** 1 IIASA - International Institute for Applied Systems Analysis, Laxenburg, Austria; 2 Institute for Environmental Decisions, ETH Zürich, Zürich, Switzerland; 3 Deltares, Delft, Netherlands

**Keywords:** Fiscal Risk, Caribbean Region, CCRIF, Climate Storylines, Panel Regression, 62J, 86A08, 91G70, 91B05, C23, D81, G28, H11, Q54

## Abstract

Fiscal resilience against disasters is vital for the recovery in the aftermath of climate hazards. Without swift access to available funds for disaster relief, damages to human and the economy would be further exacerbated. How insurance may influence fiscal performance over time and can increase fiscal resilience for today and under a future climate has not been looked at yet in detail. Focusing on the Caribbean region and on the fiscal performance of governments after disaster events, we empirically analyze the effectiveness of the Caribbean Catastrophe Risk Insurance Facility (CCRIF) regarding the reduction of short-term fiscal effects. We embed this analysis within a novel climate impact storyline approach where we produce past plausible events and investigate the usefulness of insurance under such events. The storylines were modified according to global and climate change related boundary conditions to address the issue whether the CCRIF is fit for purpose or will need to be adapted in the future. We found that both hurricane strikes and the CCRIF affect fiscal outcomes of Caribbean countries. Furthermore, there are indications that CCRIF can counteract the negative fiscal consequences over the short term period induced by the disaster. Our analysis should shed some light on the current discussions on how development related assistance can be structured to enhance climate resilience in highly exposed countries for both direct and fiscal impacts of disasters.

**Supplementary Information:**

The online version contains supplementary material available at 10.1007/s41885-023-00126-0.

## Introduction

Natural hazards as well as corresponding damages are still increasing all around the world (Munich Re 2018). Besides increasing assets in hazard prone areas, climate change is already contributing to the mounting damages of disasters (IPCC 2018, IPCC 2022). While considerable attention has been given in the past to the direct damages caused by natural hazards, indirect impacts, i.e., losses that occur due to direct damages (Hallegatte et al. 2007), and the management of it, are gaining increasingly attention (Reichstein et al. 2021). This is not to the least because in our interconnected world, direct losses can easily spread across different sectors and risk bearers and therefore can cause large opportunity costs (Centeno et al. 2015; World Bank 2021) including hindering sustainable development (Hallegatte et al. 2020). Global agendas and frameworks such as the Paris Agreement, the Sustainable Development Goals as well as the Sendai Framework for Risk Reduction are therefore calling for the explicit inclusion of such risk dimensions, usually subsumed under the term resilience (UN 2015; UNDRR 2015; Noy and Yonson 2018).

The government is usually seen as one key risk bearer, both for public and private losses, and is expected to be responsible to assist and support a fast recovery of the economy (Hochrainer-Stigler et al. 2018). Therefore, fiscal resilience is an important dimension as urgent liquidity is vital for humanitarian aid and the recovery process as without swift access to available funds for disaster relief, damages to human and the economy would be further exacerbated because of lacking necessary logistics and slow recovery (World Bank 2021). To enhance fiscal resilience various options are available to governments, including setting up reserve funds, contingent credits, emergency loans or insurance (Ghesquiere and Mahul 2010), particularly in the form of sovereign risk pools (Ciullo et al. 2023). While current approaches usually assess such management options using the full probabilistic information about all possible disaster events that could happen (Woo 2011) we introduce in this paper a so-called climate storyline approach (van den Hurk et al. 2023). In essence, climate storylines are based on past events but include plausible different risk realizations. Furthermore, these climate storylines can be assessed under different future climate and socio-economic settings. One of the strengths of such an approach compared to a probabilistic assessment is that it improves risk awareness as it reproduces disaster events that are similar to those already experienced and thus can be personally related to (Shepherd et al. 2018). Such climate storylines can therefore trigger a wake-up call and motivation to change and improve climate risk governance (Krauß and Bremer 2020). Another strength of such an approach compared to probabilistic ones is in regard to climate change considerations as top-down approaches from global climate model ensembles simulations are usually inadequate in dealing with uncertainties in regional climate predictions as aleatoric and epistemic uncertainties are mixed (Shepherd et al. 2018). By focusing on climate storylines no such probabilistic information is needed and consequences can be assessed and understood conditioned on specified climatological and socio-economic boundary conditions (van den Hurk et al. 2023).

Recognizing the potential benefits of a climate storyline approach we developed and applied a corresponding framework to the Caribbean and Central America (CCA) region to analyze the fiscal performance of local governments due to cyclone risk and potential benefits of insurance. The area was chosen as it is one of the most vulnerable regions to tropical cyclones and saw the establishment of the first multi-country risk pool in the world, namely the Caribbean Catastrophe Risk Insurance Facility (CCRIF). CCRIF was designed as a regional catastrophe fund for local governments to limit the financial impact of devastating hurricanes and earthquakes by quickly providing financial liquidity when a policy is triggered. We therefore specifically focus on possible liquidity gaps of governments within the Caribbean region, i.e. not having enough resources to address all emergency related losses, their consequences on fiscal performance over time and the effect of CCRIF on these fiscal effects. Up to now, an empirical analysis of such pooling arrangements to tackle fiscal risks was not looked at yet. We relate these results with climate storylines for the year 2016 and 2017 and analyze fiscal performance over time for those events. In a final step, we include climate and global change scenarios and investigate future changes in fiscal performance under these new boundary conditions. We found that fiscal effects can be significant due to natural hazard events for Caribbean countries and there are indications that insurance can help to mitigate some of these fiscal effects in the short term. The climate storylines showed that many more devastating events (in terms of damages, number of countries hit, number of consecutive events) could have happened in the past, and consequently fiscal stress in the region could have been much higher under such plausible events, with some of the fiscal stress reduced through the participating countries in CCRIF.

The paper is organized as follows. The “Context and Case Study Area” section presents a discussion on the liquidity gap concept, its relation to insurance instruments and more specifically to the CCRIF to set up the stage for the “Methodology” section, including a detailed discussion on the climate impact storyline and the panel regression approach used. The “Results” section then presents the corresponding results and afterwards the “Discussion” section discusses them within a broader development and climate resilience context. Finally, the “Conclusion” section summarizes our findings and provides an outlook to the future.

## Context and Case Study Area

### Liquidity Gap

**Fig. 1:**
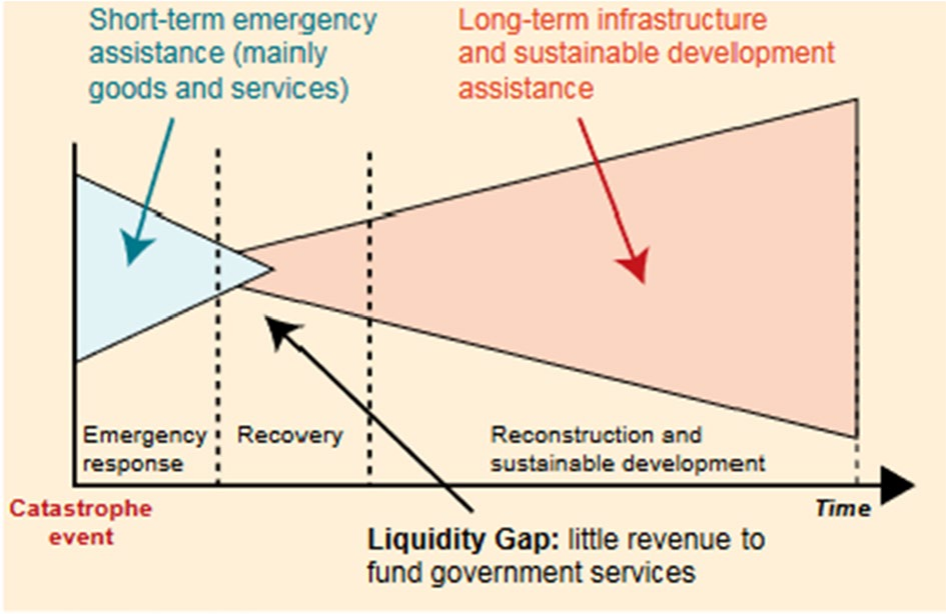
The liquidity gap and the different phases of recovery (Young 2010)

Simplistically speaking but serving our needs (for various other approaches we refer to the recent IPCC report 2022) after a natural disaster one can distinguish three different phases of needs and priorities over time, including 1) an emergency response phase where short-term emergency assistance is needed (usually in the form of goods and services), 2) a medium term recovery phase to restore the basic functioning of the affected area and a 3) long-term reconstruction and sustainable development phase which includes infrastructure and sustainable development assistance (Fig. 1). For all of these phases, different instruments may be more appropriate than others (see for a discussion Mahul et al. 2019; Mochizuki et al. 2019). Very often the international community provides ex-post relief, but such funds are slow to mobilize (often taking 4–12 months) and are not always efficiently used. Government borrowings and budget reallocations also take some time. Smaller countries such as those in the Caribbean and most SIDS (Small Island Developing States) with high debt burdens cannot afford to self-finance disaster risk or rely solely on ex-post financing strategies such as debt relief which are invariable loans, or using their own limited budgets through budget reallocations or taking resources away from funded projects and programmes to support recovery and reconstruction. Therefore, more proactive instruments that are set-up before extremes events occur are needed in such cases (Mahul et al. 2019).

For the medium-term recovery phase and the possibility of a liquidity gap, i.e. having not enough resources to finance losses the government is responsible for, for example due to little revenue to fund government services, insurance (as in the case of Caribbean countries) or catastrophe bonds (as in the case of Mexico) are feasible options to reduce such risk. The term liquidity gap is not unique and other terms are also introduced in the relevant literature including financing gap or shortfall or fiscal gap (Hochrainer 2006) among others. The underlying idea, however, is the same, i.e. losses an agent is responsible for cannot be financed with its resources at hand. We use the term liquidity gap as it was specifically introduced for the CCRIF which is one of our major focal points in this paper. The Caribbean Catastrophe Risk Insurance Facility (CCRIF) was established in 2007 as a regional mechanism to contain the fiscal costs of disasters and to bridge the liquidity gap in the immediate aftermath (CDKN 2012). Essentially, what CCRIF does is to fill that post disaster liquidity gap and it supports its member governments to help their populations, businesses and key sectors such as education and agriculture after a disaster. This is discussed in more detail in next section.

### The Caribbean Catastrophe Risk Insurance Facility (CCRIF)

The idea for CCRIF was born as a response to Hurricane Ivan in 2004, which caused billions of dollars of losses across the Caribbean (detailed information as given below can be found at https://www.ccrif.org). For example, losses were close to 200 percent of the annual GDP for Grenada as well as the Cayman Islands. Following the passage of Ivan, the Caribbean Community (CARICOM) Heads of Government approached the World Bank for assistance to design and implement a risk financing mechanism to support member governments and provide quick liquidity in the aftermath of disasters. This was the start of what would become the Caribbean Catastrophe Risk Insurance Facility. It was launched in 2007 as the world`s first multi-country, multi-peril risk pool, providing parametric insurance coverage for tropical cyclones and earthquakes to 16 Caribbean governments.

Parametric insurance products are insurance contracts that make payments based on the intensity of an event (for example, hurricane wind speed, earthquake intensity, volume of rainfall) and the amount of loss is calculated beforehand. Therefore, pay-outs can be made very quickly after a hazard event. This is different from traditional insurance settlements that require an on-the-ground assessment of individual losses after an event before a payment can be made. CCRIF therefore has been designed to provide quick liquidity once a country’s parametric insurance policy is triggered. As already indicated before, the Facility is not designed to cover all losses on the ground but to ensure that governments have resources available to meet their most pressing needs after a natural disaster and avoid a liquidity gap, i.e. it was not designed to assist long term reconstruction efforts. We will therefore focus to up to one year after the disaster event (see “Storylines under Climatic and Socio-economic change” for more details).

For CCRIF the parametric insurance disburses funds based on the occurrence of a pre-defined level of hazard and impact. For an actual CCRIF policy and coverage selection, countries must make three key decisions. They have to select an attachment point which can be described as the severity of an event and corresponding loss value which gives rise to a payment. Only if the modelled loss for an event equals or exceeds the attachment point specified in the contract a payment will occur. In other words, the country covers all losses below the attachment point. Furthermore, an exhaustion point must be chosen by the country. This is the severity of the event loss at or above which the maximum payment is triggered. Consequently, all losses above the exhaustion point are retained by the country. Finally, the coverage limit needs to be selected (or the ceding percentage) by a country. The coverage limit is the difference between the attachment and exhaustion points multiplied by the ceding percentage. The later is the amount of risk between the attachment and exhaustion points that the country is transferring to CCRIF. Hence, only part of the total losses between the two points will be covered by CCRIF which is determined by the ceding percentage. It must be noted that the actual coverage selection by individual countries is not publicly available but essentially follows the procedure as explained above.

Since its inception in 2007 till 2022, CCRIF has made 58 pay-outs totalling US$260 million to its member governments all within 14 days after an event. A rough assessment of the beneficiaries of CCRIF’s pay-outs show that over 3.5 million people in the Caribbean and Central America benefitted directly or indirectly from these pay-outs after a hazard event (CCRIF SCP 2022). Regarding the acceptance of the CCRIF in the participating countries, while the payouts are relatively small compared to the overwhelming cost of rebuilding, all recipient governments expressed appreciation for the rapid infusion of liquidity, which they are able to use to address immediate priorities and to support the most vulnerable. CCRIF members consistently indicated that these rapid payouts are an invaluable benefit of membership. While the use and benefits of CCRIF are documented, an empirical analysis of CCRIF in regard to fiscal effects over time remains limited and are discussed next.

### Fiscal Risks due to Natural Disasters: Empirical Studies

Regarding the fiscal risks due to disaster events and possible ways to manage them, several empirical studies are available. However, most of them take a rather generic perspective on the issue of management of it and usually address fiscal consequences within a broad set of dimensions (e.g. see for an empirical example on the global scale Loayza et al. 2012 and more recently Panwar and Sen 2019 as well as Onuma et al. 2021; for a review of the literature see Cavallo and Noy 2011 and Botzen et al. 2019 which discusses modelling efforts as well). Within these dimensions quite different fiscal behaviours were empirically found, especially between developed countries and developing countries (Noy and Nualsri 2011). For example, Lis and Nickel (2010) regressed the change in government budget balance relative to GDP on direct damages reported by EMDAT (a natural disaster database). They distinguished between direct fiscal effects due to relief and reconstruction payments that enter on the expenditure side, and indirect effects due to the macroeconomic shock that may reduce tax revenue and other sources of income. The authors find that an extreme event leads to a decrease in the budget balance of 0.23% across all countries and 0.27% in low-income countries. The study by Melecky and Raddatz (2015) estimated the shocks of natural disasters to government expenditure and revenue, per capita GDP, inflation rate, and nominal interest rate. They found that developed insurance and credit markets attenuate the output shock due to disasters but in different ways. On the one hand, countries with developed credit markets can increase their budget deficit more strongly in the aftermath of a disaster than countries with less developed credit markets. On the other hand, countries with high insurance penetration rates do not increase their deficit but also do not experience the decline in growth that countries with low insurance penetration face.

In the context of the Caribbean, quite a number of studies looked at the fiscal consequences due to natural disaster events. For example, Strobl (2012) and Hsiang (2010) showed, using exogenous measures, that tropical cyclones have severe macroeconomic effects in this region. Ouattara and Strobl (2013) found that hurricanes lead to a short-term increase in government spending of countries in the region, in turn leading to lower budget balance. There are, however, no significant effects on the capital account, tax revenue, and debt. Probably most relevant to our study is Ouattara et al. (2018) which found that government revenue decreases in the month of the event, but government spending does not respond significantly. The latter is potentially related to the strong budget constraints domestically. While they use very detailed monthly data to analyse the fiscal performance over time, they did not included possible effects due to the CCRIF payouts which is one primary question in our paper. Furthermore, they covered a six-month period after the disaster and suggested that for variables such as debt or external grant, due to lack of monthly data, to extend the examination period to at least 12 months, which was the case for our analysis. Moving forward, Mohan et al. (2018) reported an increase in government consumption in the year after hurricane strikes. More recently, Mohan and Strobl (2020) analysed the effects of hurricanes on debt accumulation and composition in Caribbean and Central America. Their results indicate that public debt increases after hurricane strikes, primarily attributable to external credits to the central government, e.g., by international organizations such as World Bank and IMF. In our analysis we base and extend these results including the use of a more detailed (e.g. quarterly) dataset.

Summarizing, the aforementioned studies indicate that natural disasters significantly impact the government fiscal situation, especially revenue and expenditure, and there are indications that insurance could help reduce this negative effects. However, while existing research shows that natural disasters could damage the tax bases, induce higher expenditure and more external debt, the influence of risk instruments such as insurance still needs to be quantified. As will be discussed next, we will address these gaps by employing a panel regression analysis on a very detailed dataset to reveal the relationship between hurricane damages, CCRIF participation and fiscal performance of Caribbean and Central American governments. These results will be used afterwards for building climate impact storylines of past plausible events which indicates the usefulness of CCRIF in regard to the reduction of fiscal risk over time in the past. This analysis is then extended to include also possible climate change effects and its performance on CCRIF in the future.

## Methodology

There is an ongoing debate within the disaster risk as well as climate change research community about the most appropriate use of past and projected climate data and impact models to support decision making under deep uncertainty. For example, past data on extremes are, by nature, rare and therefore estimation of risk, based on such data, is very uncertain. Impact models of all kinds, including catastrophe modelling approaches (Woo 2019), can generate hazard events to provide a probabilistic assessment and even future increases due to climate change but are criticised to mix aleatoric (e.g. due to the nature of weather events) and epistemic uncertainty (e.g. due to lack of knowledge). The use of such models in climate adaptation decision support often relies on classic frameworks such as cost benefit analysis (especially in the case where quantitative information of risk and costs are available). However, these frameworks have various methodological limitations, e.g., in regard to setting discounting values (see Mechler 2016), and policy, e.g. results difficult to communicate to non-experts, and several authors therefore proposed an alternative solution known as climate impact storyline approach (Shepherd et al. 2018).

**Fig. 2:**
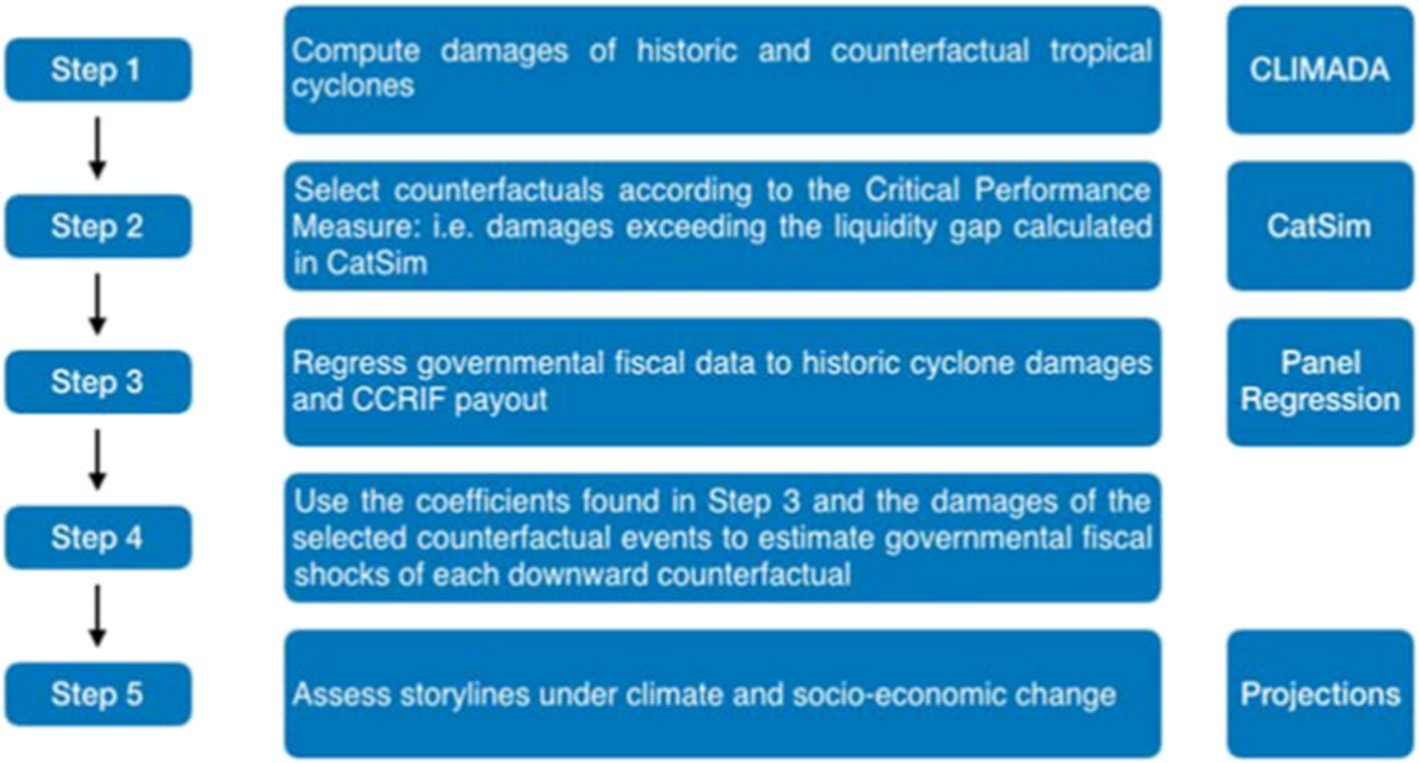
Methodological approach for assessing fiscal risks through counterfactuals

According to Shepherd et al. (2018) a storyline is defined as “a physically self-consistent unfolding of past events, or of plausible future events or pathways”. The focus here differ from classic probabilistic approaches as it focuses on past events and plausible other realizations of these events. Furthermore, climate storylines aim at describing and understanding individual climate events and possible future changes. We refer the reader to van den Hurk et al. (2023) for more details regarding the selection criteria for climate storylines including areas where they can be used, and, in particular, on their suggestion for the construction of “realistic” storylines and focus on hotspot areas. In the present work, we build climate storylines following the framework proposed by Ciullo et al. (2021) where climate storylines are derived from downward counterfactuals, i.e., from alternative versions of past events that generated a larger negative (“downward”) outcome compared to history. In this way, we use downward counterfactual thinking as part of risk analysis and management, as suggested for example by Woo (2019). A summary of our approach can be found in Fig. 2.

Essentially, we create a large set of plausible counterfactual damages from tropical cyclones for our case study area, i.e. the Caribbean region, using forecast tropical cyclones data and the CLIMADA impact modelling platform, see section “The CLIMADA impact modelling platform”. Out of this large set of counterfactual tropical cyclones, we select those which are specifically critical from a liquidity gap perspective and potential payouts from CCRIF discussed in the section “Selection of Counterfactuals”, i.e., the downward counterfactuals, which compose climate impact storylines. Storylines are finally projected in the future to include climate and global changes as explained in section “Storylines under Climatic and Socio-economic change”. Finally, how the counterfactuals are affecting fiscal risk and the usefulness of CCRIF are analyzed based on the results reported by the empirical approach where we estimated, through a detailed panel regression approach, the changes in fiscal risk indices due to past cyclone events, see section “Fiscal Risk due to Disaster Events: Data used and Panel Regression Approach”.

### Data Summary

Quarterly fiscal data of CCRIF countries were collected for the years 1997 and 2018 (see Table S1 in the Supplementary), using specific country-based and global datasets such as the World Bank and IMF and especially official government websites (see Table S2 in the Supplementary). Summarizing the following countries were included: Anguilla, Antigua & Barbuda, Belize, Barbados, Dominica, Grenada, Guatemala, Guyana, St. Kitts & Nevis, St. Lucia, Montserrat, Nicaragua, El Salvador, Suriname, Trinidad & Tobago, St. Vincent & Grenadines. Only sovereign countries were selected in the analysis with the exception of Anguilla and Montserrat, which are British overseas territories, since they are members of the Eastern Caribbean Central Bank. For those territories exchange rate and GDP deflator is assumed to equal the ones of the neighbouring Saint Kitts and Nevis. Identified fiscal variables (see Table S3 in the Supplementary) were searched for on a quarterly basis while available monthly values were aggregated to quarterly values, converted to current US dollars in order to account for exchange rate fluctuations, and deflated to 2010 prices using the WDI GDP deflator. Detailed summary of statistics of the variables as well as CCRIF payouts can be found in Table S4 as well as S5 in the Supplementary.

### The CLIMADA Impact Modelling Platform

Direct economic damages from tropical cyclones are estimated using the open-source and open-access CLIMADA impact model. We here briefly describe CLIMADA focusing on the modeling set-up implemented in the current study and refer the interested reader to Aznar-Siguan and Bresch (2019) and Bresch and Aznar-Siguan (2021). Direct damages in CLIMADA are assessed as a function of weather-related hazard, exposure of people and goods to such hazards, and vulnerability of the exposed entities. Hazard from tropical cyclones is represented in CLIMADA by a map of 1-min sustained wind gusts, modelled as the sum of two components: a static circular wind speed and a translational wind speed arising from the tropical cyclone movement. Both components are derived from information about tropical cyclone tracks such as time, location, radius of maximum winds, and central pressure (Geiger et al. 2018). CLIMADA provides various built-in methods to generate exposure (see, e.g., Aznar-Siguan and Bresch 2019; and Eberenz et al. 2020). We use the one introduced in Gettelman et al. (2018) where the exposed economic value is calculated by downscaling regional Gross Domestic Products (GDP) using nighttime lights data. Vulnerability in CLIMADA is represented, as typically done in natural catastrophe modelling, via a damage function which relates hazard intensity to damage percentage in exposed value and we use the calibrated vulnerability functions proposed by Eberenz et al. (2020). Hence, similar to the proprietary model employed by CCRIF, CLIMADA operationalizes the risk framework in which

$$\mathrm{damage}=\mathrm{f}(\mathrm{hazard},\mathrm{ exposure},\mathrm{vulnerability}) =\mathrm{exposure }\times \mathrm{f}\_\mathrm{imp }(\mathrm{hazard})$$

where f_imp_ is a vulnerability or damage function that relates physical intensity, e.g. wind speed, to mean damage degree. Historic tropical cyclone tracks are retrieved from the International Best Track Archive for Climate Stewardship (IBTrACS) dataset (Knapp et al. 2010). Counterfactual tropical cyclones tracks are simulated using forecast data provided by the Observing System Research and Predictability Experiment (THORPEX). THORPEX initiated in 2005 the THORPEX Interactive Grand Global Ensemble (TIGGE) program, which contains many forecasting data sets of tropical cyclone tracks from several international meteorological agencies. The dataset contains historical tropical cyclone track data since 2008 and is updated continuously. The resulting values from hazard, vulnerability and exposure are then aggregated to country-quarter observations for our purpose.

### Selection of Counterfactuals

Focusing on tropical cyclones, we apply CLIMADA to the case of fiscal risk assessment and management of Caribbean﻿ countries. At the beginning, a full set of all counterfactual events was built based on past events starting from the year 2007 which are relevant to our area of interest. This resulted in hundred of thousands possible plausible events. For all these tropical cyclone events, damages were estimated using CLIMADA and all events with small losses or losses which do not reach the liquidity gap were excluded (based on the CatSim approach that estimates which resources are available, see Hochrainer-Stigler 2021). The final selection of what tropical cyclones compose a storyline was made based on stakeholder interaction (e.g. the suggestion for looking at consecutive events or events that happen for more countries at once) and by looking at the most impactful or relevant counterfactual events. The two constructed storylines along with the selected tropical cyclone events are reported in Table 1.

**Table 1:** Storyline composition

Storyline name	Selected tropical cyclones	Quarter of occurrence
Extreme Quarter (EQ)	Maria	3rd quarter of 2017
	Irma	3rd quarter of 2017
	Harvey	3rd quarter of 2017
Consecutive Quarters (CQ)	Gascon	3rd quarter of 2016
	Karl	3rd quarter of 2016
	Matthew	4th quarter of 2016

**Fig. 3:**
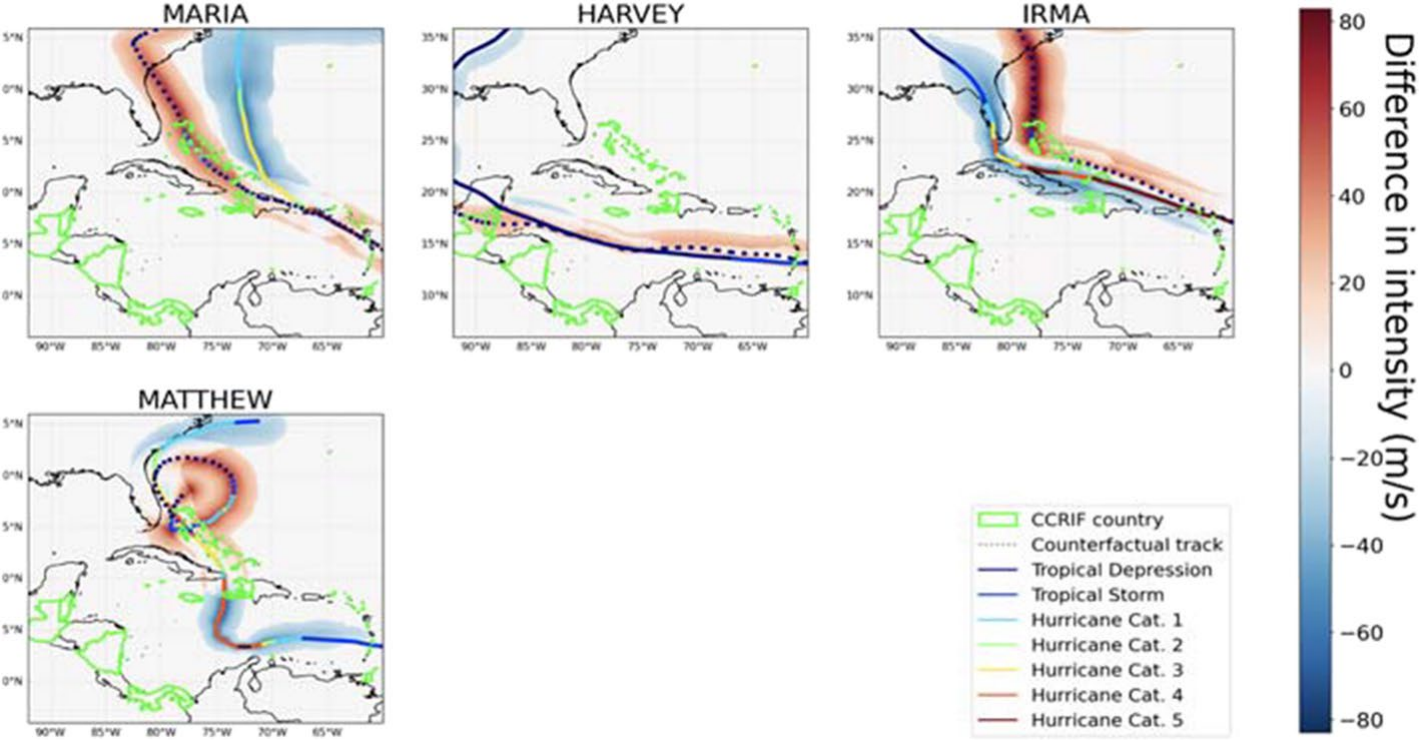
Selected historical (solid line) and counterfactual storms (dashed line) in the Caribbean region, the color shows the difference of wind speed between the counterfactual and the historical storm

The first storyline, named “Extreme Quarter” (EQ), involves downward counterfactuals of Storm Maria, Harvey and Irma in 2017. As shown in the first row of Fig. 3, the counterfactuals not only hit more countries, but also became stronger. This results in higher damages. The second storyline features Consecutive Quarters (CQ). The storm season in the Caribbean region usually covers the third quarter (July-September). Some storms occur later in the season but these are generally not very damaging. However, in October of 2016, Storm Matthew hit the region and caused losses of more than 30 billion dollars. This inspired the construction of a counterfactual storm season with two storms in the third quarter (Gaston and Karl), and one in the fourth quarter (Matthew) (Table 1). This allows us to stress test the Caribbean countries and CCRIF under consecutive shocks. The two climate storylines are finally projected under different future conditions in order to investigate the future performances of the CCRIF. This is discussed in the next section.

### Storylines under Climatic and Socio-Economic Change

When developing future storylines, we take the change of economy and climate conditions into account. Three intensity scenarios were considered: Low Intensity, Mid Intensity, as well as High Intensity. The economic and climate component of each scenario is derived from the collection of Shared Socio-economic Pathways (SSPs) and different level of cyclone intensity increase, as explained in Table 2.

**Table 2:** Change of wind speed and asset value in each future climate change scenario

Climate change scenarios	Change of cyclone intensity of selected counterfactuals	Change of asset value of all CCRIF countries
High Intensity	+ 10%	+ 450% (SSP5)
Mid Intensity	+ 5%	+ 320% (SSP2)
Low Intensity	0%	+ 260% (SSP1)

In terms of climatic perturbations, we adjusted tropical cyclone intensity following the estimation provided by Knutson et al. (2020) who stated that tropical cyclone intensity might increase between 1 to 10% in a 2 degrees warmer world. Detailed socio-climate parameter settings are given in Table 2 as well and are taken from the Socio-Economic Pathways hosted at IIASA. We ran the simulation in CLIMADA with updated exposure and storm intensity settings corresponding to each intensity scenario to calculate the damage of downward counterfactuals in future storylines.

### Fiscal Risk due to Disaster Events: Data used and Panel Regression Approach

In our analysis the liquidity gap and corresponding consequences as well as the CCRIF coverage to lessen the risk of a liquidity gap all belong to the recovery phase, i.e. shortly after the disaster event, which we have chosen to be up to 1 year (Fig. 3). Hence, yearly data of fiscal variables, which is usually used for an analysis of macroeconomic effects, would be a too rough temporal resolution for our needs while monthly data would lead to too many dependent variables to be estimated (i.e. up to 12 months after the disaster event). Therefore, quarterly macro-economic data had to be gathered instead. The detailed dataset for the Caribbean region was built through an intensive data acquisition and transformation process of macroeconomic data. Furthermore, according to prior research as described in the empirical literature review, we selected corresponding fiscal indices and focus here on revenues, expenditure and indebtedness variables through the rest of the paper.

**Fig. 4:**
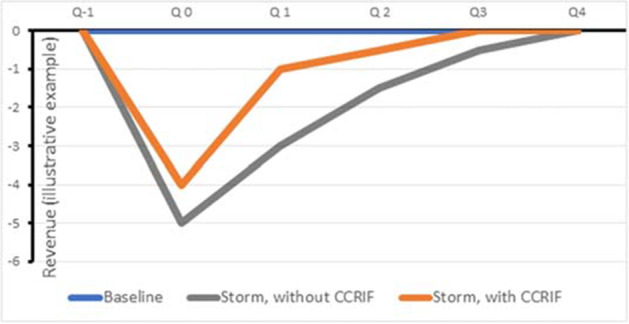
Illustration of hypothetic short-term effects of hurricanes and CCRIF and fiscal index

The use of a panel regression approach stem from the need of comparing different countries over time. To make the data comparable across countries and over time, we use the division of the quarterly fiscal indices by the GDP of corresponding year. The ratio of fiscal index over GDP should remain stable after removing the yearly trend, seasonal variations, and the national differences. Therefore, when a non-CCRIF country gets hit by a hurricane, the fluctuation of this ratio should be attributed to direct damages of the disaster. Fiscal performance over time as indicated before are effects after the disaster event. Taking government revenue as an example, as shown in Fig. 4 the grey line indicates the government lost income after the event happened. However, if this country were insured by CCRIF, not only the magnitude of shock would be reduced, but also the lasting time of the impact could be shortened, as given by the orange line. This could be interpreted as a reduction of fiscal effects.

Therefore, not only the change of fiscal indices was examined in the quarter of the event, but also changes of the quarters after the event were looked at. Out of this design, we applied a panel regression model as given in Eq. 1. Three quarterly indices were used as dependent variables to represent the government fiscal performance: revenue, expenditure and debt. They were measured in proportion to the yearly GDP. The regression model examines how much would these fiscal variables diverge from the baseline level (blue curve in Fig. 4) in the quarter of the disaster and the quarters afterwards. Furthermore, the model also tests to what extent the payout from CCRIF counteracts the negative impact on the fiscal performances of governments.

In more detail, in Eq. 1 the continuous variable damage captures the damages caused by hurricanes, measured in billion dollars, while the amount of payout is represented by CCRIF. Such design of the model compares the fiscal performance of member states before and after joining CCRIF time wise. Additionally, it checks the difference of performance between non-member states and those which were supported by CCRIF. In total the fiscal data of 16 countries were collected, among which 6 never received payout from CCRIF.

1 $$\frac{{fiscal\_\mathrm{var}}_{i,\mathrm{t}}}{{\mathrm{GDP}}_{\mathrm{i},\mathrm{t}}}=\rho \bullet {\frac{{\mathrm{fiscal}\_\mathrm{var}}_{\mathrm{i},\mathrm{t}-1}}{{\mathrm{GDP}}_{\mathrm{i},\mathrm{t}-1}}+ {\alpha }_{i}}+\sum_{q=0-4}{\upbeta }_{1}^{\mathrm{q}}\bullet \frac{{\mathrm{Damage}}_{\mathrm{i},\mathrm{t}-\mathrm{q}}}{{\mathrm{GDP}}_{\mathrm{i},\mathrm{t}-\mathrm{q}}}+\sum_{q=0-4}{\upbeta }_{2}^{\mathrm{q}}\bullet \frac{{\mathrm{CCRIF}}_{\mathrm{i},\mathrm{t}-\mathrm{q}}}{{\mathrm{GDP}}_{\mathrm{i},\mathrm{t}-\mathrm{q}}}+\gamma {Q}_{i}+\lambda {Q}_{i,\mathrm{t}}+\theta Y_{t}+{\varepsilon }_{i,\mathrm{t}}$$

It is standard procedure in such panel regression models for analyzing disaster effects to introduce other variables and remove the yearly trend and seasonality of the data. *Q* are country-specific quarter dummies to account for the seasonality in government finance. *λQ* is a country-specific linear deterministic time trend, θ*Y* are year fixed effects that capture unobservable trends that may affect fiscal outcomes globally, for instance the financial crisis of 2008 and finally ε is the error term. Robust standard errors were used to estimate the various parameters based on Maximum Likelihood Techniques. The estimated parameters were then used as input to the storylines developed and explained already above.

## Results

We follow the structure of the methodology section and start with the selected storylines described above. Table 3 shows the timing and total damage of the two selected storylines under current climate and RCP8.5. For example, in the EQ storyline, 95.9% of the total damage (57.6 billion $) is caused in the Bahamas, which is equivalent to around 30% of the total assets being destroyed. When climate change is added, the damage of the EQ storyline increases by another 50%.

**Table 3:** Damages of the two selected storylines under current climate and *RCP8.5*

Historical or Storyline	Time	Damage (billion $)
Historical	3^rd^ Quarter 2017	12.87
	3^rd^ Quarter 2016	0
	4^th^ Quarter 2016	31.88
EQ (current climate conditions)	Based on 3^rd^ Quarter of 2017	60.04
CQ (current climate conditions)	Based on 3^rd^ Quarter 2016	6.66
	Based on 4^th^ Quarter 2016	64.24
EQ (10% intensity increase)	Based on 3^rd^ Quarter 2017	89.51
CQ (10% intensity increase)	Based on 3^rd^ Quarter 2016	10.58
	Based on 4^th^ Quarter 2016	82.69

Next, in Table 4 we are showing the results of our panel regression based on the historical data (see Step 3 in Fig. 2). The change of fiscal indices (columns 3, 4 and 5) due to the direct damages (second row) of storm events and the CCRIF payout (third row) is measured at each quarter after the event. As one can see, the impact of hurricanes could be observed not only in the quarter of the disaster event, but also afterwards. This also applies to the effect of CCRIF.

**Table 4:** Relative change of fiscal indices comparted to non-disaster quarter. Direct damage is shown as fraction of the direct hurricane damage, CCRIF payout indicators as fraction of total payout. Highly significant on the 0.001 percent level marked as ***, and on the 0.01 percent level marked as **, and 0.05 percent level marked as *. Robust standard errors in parentheses

VARIABLES	Impact at different time	Current revenues	Newly raised debt via external grant	Current expenditure
Direct damage	the quarter of event	-0.0151	0.0144**	-0.0181**
		(0.00759)	(0.0039)	(0.0055)
	1st quarter after event	-0.0116	0.0401*	0.0131**
		(0.011)	(0.0134)	(0.00433)
	2nd quarter after event	-0.0102*	0.0277**	-0.0250**
		(0.00352)	(0.00707)	(0.0055)
	3rd quarter after event	0.00339	0.0297**	-0.0119***
		(0.00715)	(0.00829)	(0.00195)
	4th quarter after event	0.00923	0.0206**	-0.0061
		(0.011)	(0.00521)	(0.00466)
CCRIF payout	the quarter of event	0.317	-0.641***	0.491**
		(0.238)	(0.129)	(0.138)
	1st quarter after event	-0.00845	-1.502**	-0.152
		(0.36)	(0.431)	(0.159)
	2nd quarter after event	0.353*	-1.055***	0.787***
		(0.12)	(0.255)	(0.111)
	3rd quarter after event	1.986***	-1.143***	0.945***
		(0.216)	(0.286)	(0.0786)
	*4th quarter after event*	-0.935*	-0.848***	-0.0785
		(0.392)	(0.199)	(0.168)
*Lagged fiscal variable*		0.461***	*0.00369*	0.347*
		*(0.0652)*	*(0.0258)*	*(0.129)*
*Constants*		0.507***	0.796**	0.821***
		*(0.0543)*	*(0.258)*	*(0.151)*
Observations		1,050	888	1,052
R-squared		0.671	0.172	0.642
Number of countries		14	13	14

It is worth noting that in our model, both the dependent variables (fiscal indices) and the independent variables (hurricane damages, and CCRIF payout) are normalised by GDP. Therefore, for datapoints of the same year, these variables share the same denominator. We therefore neglect the common denominator, which in our case is the annual GDP when interpreting the results.

As highlighted in Table 4, the government’s grant/debt increases by 0.0144 USD in the quarter of the hurricane. Two quarters later, the debt further increases by 0.0277 USD, and the revenue will decrease by 0.0102 USD compared to the non-disaster baseline level as illustrated by the blue curve in Fig. 4.

CCRIF payout is intended to counteract the impact of disaster damages which is also empirically validated in our analysis. For example, in the second quarter after the event, CCRIF member states’ income increased by 0.353 USD per dollar CCRIF payout. The effect of CCRIF on revenue is more significant in the third quarter after the event, where revenues due to CCRIF payouts are nearly doubled. This indicates that the payout of CCRIF not only functions as an extra source of money, but also is indirectly beneficial for the selected fiscal indices. For example, as was hypothesized (see Young 2010) that the money from CCRIF initiates disaster relief and also increases the tax base for the government. A similar mechanism applies to debt. Per dollar payout from CCRIF, the total debt of the member states accumulated over the first year after the disaster is 5.189 USD smaller than without insurance. The coefficients of payout are much larger than those of damages, but the net fiscal effect is dominated by the direct damages, which can be 100 times larger than the CCRIF payout. Therefore, when a disaster happens, the fiscal variables are still negatively affected, but also compensated by indirect responses to the CCRIF payouts.

In our last step, the storyline results and panel regression results are combined. Multiplying the damage from each storyline with the coefficients in Table 4, one can assess whether and to what extent the government’s debt would increase compared to the history (as illustrated by the baseline in Fig. 4). This leads to a total $ 4.2 billion increase of debt under the Extreme Quarter storyline compared to history over a year since the event, as given in Table 5. In order to counteract this amount of additional increase in debt and balance the debt to historical level (i.e. from the gray line back to the orange line in Fig. 4), a payout of around $ 1.2 billion is needed from CCRIF, which would bring about a total revenue increase of $ 0.2 billion compared to the non-disaster baseline level (blue line in Fig. 4). This rise in revenue is predictable since CCRIF payout plays an important role as extra income for the government. However, this amount of payout is far beyond CCRIF’s capacity (and any written contract), indicating that in face of unprecedented large events, CCRIF will not be sufficient to stem the fiscal performance to the historical level and other instruments probably would need to be set up instead, e.g. risk reduction.

**Table 5:** Damage, payouts and fiscal impacts of the “Extreme Quarter” storyline (in bn. USD). CCRIF payout in the EQ storyline is calculated so that the total debt change over a year remains the same as the historic level

	Direct impact	Calculated change of debt in each period					CCRIF payout	Total change of fiscal variables over a year since the disaster	
		Quarter of event	1st quarter after event	2nd quarter after event	3rd quarter after event	4th quarter after event		Debt	Revenue
History	12.9	0.2	0.5	0.3	0.3	0.2	0.035	1.5	-0.3
Cf without CCRIF	60.0	0.9	2.4	1.7	1.8	1.2	0.0	4.2	-1.5
EQ	60.0	0.1	0.5	0.4	0.4	0.2	1.2	1.5	0.2

Moving on to the second storyline “Consecutive Quarters” the situation is similar to the “Extreme Quarter” storyline and results are shown in Table 6. The counterfactual storm of Matthew in October causes in total 64.2 billion USD damages in the Bahamas. Counterfactuals of Storm Gaston and Karl as given in Table 3, which happened in the first quarter of the CQ storyline, cause a $ 6.7 billion damage in the region. To balance this extra shock to the debt level caused by these two consecutive disaster quarters, countries hit by the storm require in total $ 0.92 billion from CCRIF ($ 0.12 billion for the First Disaster Quarter in CQ and $ 0.8 billion for the Second Disaster Quarter in CQ). The situation becomes worse under future climate conditions, as shown in Table 6. Climate change increases the wind speed of storms, hence causing more damages. The disaster impact further increases when considering economic growth in different SSPs. However, considering the scale up of economy, CCRIF may also increase correspondingly its pooling size because of the economic growth. Be as it may, the need to increase funding size of the CCRIF in the future seems to be necessary to address increases in losses.

**Table 6:** Payout needed in different storylines to balance the extra impact of intensified storm on revenues, unit: billion USD

History or storylines		Damage	Payout/Payout needed	Revenue change over a year	Debt change over a year
3^rd^ Quarter of 2017		12.9	0.035	-0.3	1.5
3^rd^ Quarter of 2016		0	0	0	0
4^th^ Quarter of 2016		31.9	0.006	-0.8	4.2
EQ	Current climate	60.0	1.2	0.2	1.5
	Low Intensity	216.1	5.2	1.9	1.5
	Mid Intensity	310.3	7.63	2.9	1.5
	High Intensity	491.4	12.3	4.8	1.5
CQ (First Disaster Quarter)	Current climate	6.7	0.12	0.1	0.0
	Low Intensity	24.0	0.6	0.3	0.0
	Mid Intensity	35.6	0.9	0.4	0.0
	High Intensity	58.1	1.5	0.6	0.0
CQ (Second Disaster Quarter)	Current climate	64.2	0.8	-0.5	4.2
	Low Intensity	231.3	5.1	1.3	4.2
	Mid Intensity	309.8	7.1	2.1	4.2
	High Intensity	454.0	10.8	3.7	4.2

We conclude from our analysis that cyclone events in the Caribbean can cause short-term fiscal impacts. Furthermore, there are indications that CCRIF had played an effective role in reducing the fiscal shocks over time (or indirect risks over time) in the participating countries and would have been even more so under the considered storylines, i.e. even more useful under plausible past events. Note, the fund will, nevertheless, be insufficient under assumed future climate conditions due to the large increase in damages. It is therefore highly likely that CCRIF will needs to increase its capitalization to keep pace with the economic growth in the area and increases in cyclone risk due to climate change. It should be noted that in our future storylines, due to the very large economic damage the fiscal shocks can become unbearable and the CCRIF alone would not be useful as it is solely a recovery instrument and additional measures would need to be take place, including risk reduction efforts. Risk reduction and risk financing instruments can enhance climate resilience and our results also have important implications for development assistance as will be discussed next within a broader context.

## Discussion

According to our analysis fiscal effects can be significant due to natural hazard events for Caribbean countries and there are indications that insurance can help to mitigate some of these fiscal effects in the short run. As a consequence, especially from a humanitarian perspective and for enhancing the governmental fiscal resilience such an instrument is worthwhile to be considered as one possible option for today and in the future, not only for this case study but also more generally. Indeed, fast recovery may be even more important in regard to compound risks (Zscheischler et al. 2020) where some risk realization in one dimension also has significant consequences on other risk realizations. For example, the COVID-19 pandemic showed quite well how fiscal stimulus packages can influence other instruments that may support fiscal resilience against natural disaster risks (Hochrainer-Stigler 2021; Chang et al. 2022). Furthermore, COVID-19 especially made already quite vulnerable countries such as the ones in CCA even more vulnerable to natural disasters and therefore need additional outside support.

However, there are several limitations of our approach which should be kept in mind. The use of the catastrophe model CLIMADA hindered us to account for changing exposure over time and therefore past losses are overestimated within the panel regression. While there would have been the possibility to use actual losses, such as given by disaster databases like EMDAT, it would have not been consistent with the overall approach of linking the panel regression results with the selected climate storylines through CLIMADA. In regard to the payments through CCRIF we only used past payouts in the panel regression while it is essentially the case that for each country specific trigger conditions were set dependent on their fiscal situation. This means that for different countries different attachment, exhaustion points and coverage limits have to be assumed but were not included in the analysis due to lack of information. In the future, if such information would be available a clear link between damages and payouts could be established and therefore also different policy options in terms of contract designs analyzed within a storyline approach. In that regard, another omitted topic due to the unavailability of the country insurance policies is basis risk, i.e. the risk that losses occur but insurance is not triggered and therefore no payments are made. In other words, basis risk should be reduced as much as possible, and the CCRIF recently included new catastrophe models to reduce such kind of risks. Still, due to the parametric insurance scheme basis risk cannot be fully eliminated and therefore need to be taken into account in future considerations about fiscal risk management options to reduce liquidity gaps. Last but not least, due to lack of data we did not included an analysis of the risk of insolvency of CCRIF which, while unlikely, is a possibility under some extreme scenarios.

CCRIF could not have been established without outside support and therefore deserves a discussion within our context. For example, a well-functioning CCRIF is closely connected with the global responsibility of the European Union in enhancing climate resilience in vulnerable countries. The insurance mechanism was funded by a two Multi-Donor Trust Funds (MDTF) in 2007 and 2012 under the supervision of the World Bank. However, both EU member states and the European Commission have participated in the MDTFs. From the European perspective it is thus relevant whether these quasi-fiscal transfers to establish the CCRIF have resulted in an institution that is effective in mitigating the adverse fiscal outcomes for LAC countries. In particular, the CCRIF may reduce the reliance on grants from other countries and international financial institutions such as IMF in the aftermath of climate-related shocks. For European countries and institutions but for other countries as well such transfers can be seen as contingent moral obligations (Mechler and Hochrainer 2014). As a case in point, Bermuda, Canada, France, Ireland, the United Kingdom, the Caribbean Development Bank (CDB), the European Union (EU), and the World Bank have contributed approximately $67.4 million to support CCRIF’s initial capital and operating costs. The EU and some member states had also contributed over 25% of the initial capital of the Caribbean catastrophe risk insurance facility (CCRIF). Additionally, the European Commission and the World Bank signed 2016 a Euro 14 million agreement to be executed by the Multi-Donor Trust Fund (MDTF) to facilitate access to low cost, high quality catastrophe risk insurance for the governments of Central American countries and the Dominican Republic. In 2020 to support Caribbean governments whose social and economic sectors have been significantly disrupted by COVID-19, the European Union (EU) under its Global COVID-19 Response, has provided a grant of €10 million (US$11 million) to CCRIF for premium support or for increasing coverage for its Caribbean members. This financial assistance to CCRIF was channelled through the EU Regional Resilience Building Facility managed by the Global Facility for Disaster Reduction and Recovery (GFDRR) and The World Bank to manage an uncertain future.

Not only the future is uncertain, also the past was. As shown in the two storylines, the damages of downward counterfactual hurricanes could be overwhelmingly high and the fiscal consequences due to the high losses in the storylines could be decreased significantly. The benefit of using a storyline approach is to show in a more concrete manner the advantages of tools rather than quite abstract cost-benefit analysis as the concept of probability is not used but rather the concept of plausible past events. The latter has to be emphasized as it is not merely a scenario out of the blue but is linked to events that have occurred but could have occurred differently (van den Hurk et al. 2023). In that regard it reduces complexity of translating benefits and consequences into layman’s terms while still being accurate (see also Ciullo et al. 2021). Additionally, the inclusion of climate change shows more directly the consequences of changes in intensity or frequency of risk and therefore can be related to the current situation and the need to act (Shepherd et al. 2018).

## Conclusion

It can be noted that the explicit management of indirect risk (where fiscal performance over time is a subset) is a rather new field emerged from the experience of increasing cascading events (Reichstein et al. 2021) as well as compound and systemic risks (Zscheischler et al. 2020, Hochrainer-Stigler 2020). As discussed here, traditional risk instruments as insurance may be beneficial for mitigating such risks as well, however, our analysis also indicated that future losses may increase significantly and therefore risk-reduction options need to be thought of in conjunction. Especially in the case of the Caribbean, more drastic measures in the future may need to be considered as well, such as voluntary displacement (Desai et al. 2021). New approaches such as the adaptation pathways could help in that regard to navigate through possible adaptation limits and tipping points (Schlumberger et al. 2022) and a storyline approach may be helpful as it reduces the complexities involved in such processes to a manageable level (van den Hurk et al. 2023). This should not be seen as a call for abandoning risk approaches but rather a call for new ways forward on how to combine different approaches that can shed some light on specific challenges from new angles. Especially due to the complexities involved in the assessment and management of disaster risks under compound and systemic events, a toolbox based approach within an iterative setting may seem promising to quickly react to emerging threats as well as for decreasing the complexities for stakeholders involved to appropriate and manageable levels.

## Supplementary Information

Below is the link to the electronic supplementary material.

Supplementary file1 (DOCX 32 KB)

## Data Availability

The data sources that support the findings of this study can be found in the Supplementary and corresponding datasets are available from the corresponding author, upon request.
