# Supplementary material for: Differential Fiscal Performances of Plausible Disaster Events: A Storyline Approach for the Caribbean and Central American Governments under CCRIF
Source: Econ Disaster Clim Chang. 2023 May 11:1–21. Online ahead of print. doi: 10.1007/s41885-023-00126-0 (PMC10172055; doi:10.1007/s41885-023-00126-0)
Supplement: Supplementary file 1 — Supplementary file1 (DOCX 32 KB) [file 41885_2023_126_MOESM1_ESM.docx]

**Supplementary Information**

**Table S1: Data Sample Periods for All Countries**

| country | iso3 | Sample period | If joined CCRIF |
| --- | --- | --- | --- |
| Anguilla | AIA | 2000-2018 | Yes, 2007 |
| Antigua & Barbuda | ATG | 2000-2018 | Yes, 2007 |
| Belize | BLZ | 1990-2018 | Yes, 2007 |
| Barbados | BRB | 1995-2018 | Yes, 2007 |
| Dominica | DMA | 2000-2018 | Yes, 2007 |
| Grenada | GRD | 2000-2018 | Yes, 2007 |
| Guatemala | GTM | 1995-2018 | Yes, 2012 |
| Guyana | GUY | 1999-2018 | No |
| St. Kitts & Nevis | KNA | 2000-2018 | Yes, 2007 |
| St. Lucia | LCA | 2000-2018 | Yes, 2007 |
| Montserrat | MSR | 2000-2018 | Yes, 2007 |
| Nicaragua | NIC | 2012-2018 | Yes, 2007 |
| El Salvador | SLV | 1994-2018 | No |
| Suriname | SUR | 2006-2018 | No |
| Trinidad & Tobago | TTO | 1991-2018 | Yes, 2007 |
| St. Vincent & Grenadines | VCT | 2000-2018 | No |
|  |  |  |  |
|  |  |  |  |
|  |  |  |  |
|  |  |  |  |
|  |  |  |  |
|  |  |  |  |
|  |  |  |  |
|  |  |  |  |
|  |  |  |  |

**Table S2: Data Sources used for Panel Regression**

| Country Name | URL for data extraction |
| --- | --- |
| Anguilla | https://eccb-centralbank.org/statistics/fiscals/comparative-report/4 |
| Antigua & Barbuda | https://eccb-centralbank.org/statistics/fiscals/comparative-report/4 |
| Bahamas | https://www.centralbankbahamas.com/statistics_dos.php?cat=121 |
| Barbados | http://www.centralbank.org.bb/research-publications/statistics |
| Belize | https://www.centralbank.org.bz/rates-statistics/government-finance |
| Bermuda | https://www.gov.bm/statistical-publications |
| Cayman Islands | https://www.cima.ky |
| Dominica | https://eccb-centralbank.org/statistics/fiscals/comparative-report/4 |
| Grenada | https://eccb-centralbank.org/statistics/fiscals/comparative-report/4 |
| Haiti | https://www.brh.ht/statistiques/finances-publiques/ |
| Jamaica | http://www.boj.org.jm/statistics/econdata/stats_list.php?type=8 |
| Montserrat | https://eccb-centralbank.org/statistics/fiscals/comparative-report/4 |
| St. Kitts & Nevis | https://eccb-centralbank.org/statistics/fiscals/comparative-report/4 |
| St. Lucia | https://eccb-centralbank.org/statistics/fiscals/comparative-report/4 |
| St. Vincent & Grenadines | https://eccb-centralbank.org/statistics/fiscals/comparative-report/4 |
| Trinidad and Tobago | https://www.central-bank.org.tt/statistics/data-centre |
| Turks & Caicos Islands | https://gov.tc/stats/ |
| Nicaragua | https://www.bcn.gob.ni/estadisticas/finanzas_publicas/finanzas/index.php |
| Panama | https://www.inec.gob.pa/ |
| Guatemala | http://banguat.gob.gt/inc/main.asp?id=2261&aud=1&lang=2 |
| El Salvador | https://www.bcr.gob.sv/bcrsite/?cat=1000&lang=en |
| Honduras | https://www.bch.hn/eng/sector_fiscaleng.php |
| Costa Rica | https://www.bccr.fi.cr/ |
| Guyana | https://statisticsguyana.gov.gy/data/data-tables/ |
| Suriname | https://www.cbvs.sr/en/statistics/macroeconomic-statistics/macro-economic-tables |
| Cuba | http://www.onei.gob.cu/ |
| Colombia | https://www.banrep.gov.co/en/statistics/public-finance |
| Venezuela | http://bcv.org.ve |
| Puerto Rico | World Bank Indicators, IMF annual data |
| Guadeloupe | World Bank Indicators, IMF annual data |
| Martinique | World Bank Indicators, IMF annual data |
| Saint Barthélemy | World Bank Indicators, IMF annual data |
| Saint Martin | World Bank Indicators, IMF annual data |
| French Guiana | World Bank Indicators, IMF annual data |
| Aruba | https://www.cbaruba.org/cba/do/getPage/page/statistical-monthly-tables.html |
| Sint Maarten | https://www.centralbank.cw/statistics-reporting/statistical-tables |
| Curaçao | World Bank Indicators, IMF annual data |
| British Virgin Islands | World Bank Indicators, IMF annual data |
| General | https://en.wikipedia.org/wiki/Central_banks_and_currencies_of_the_Caribbean |

**Table S3: Extracted variables for Analysis of Fiscal Effects**

| 1 Revenues and Grants | 2 Expenditures and Net Lending |
| --- | --- |
| 1.1 Current Revenues | 2.1 Current Expenditures |
| 1.1.1 Tax Revenues (nets) | 2.1.1 Wages and Salaries |
| 1.1.2 Non-tax Revenues | 2.1.2 Goods and Services |
| 1.1.3 Transfers from Public Enterprises | 2.1.3 Interests |
| 1.1.4 Transfers from Public Financial Institutions | 2.1.4 Transfers to: |
|  | 2.1.4.1 Rest of General Government |
|  | 2.1.4.2 Public Enterprises |
|  | 2.1.4.3 Financial Public Institutions |
|  | 2.1.4.4 Private Sector |
|  | 2.1.4.5 Rest of the World |
| 1.2 Capital Revenues | 2.2 Capital Expenditures |
|  | 2.2.1 Gross Investment |
|  | 2.2.2 Transfers to: |
|  | 2.2.2.1 Rest of General Government |
|  | 2.2.2.2 Public Enterprises |
|  | 2.2.2.3 Financial Public Institutions |
|  | 2.2.2.4 Private Sector |
| 1.3 Grants | 2.3 Net Lending |
|  | 2.3.1.1 Rest of General Government |
|  | 2.3.1.2 Public Enterprises |
|  | 2.3.1.3 Financial Public Institutions |
|  | 2.3.1.4 Private Sector |

| Debt components |  |  |
| --- | --- | --- |
| 1 Total Public Debt | **2 External Public Debt** | **3 Internal Public Debt** |
| 1.1 Non-Financial Public Sector | 2.1 Non-Financial Public Sector | 3.1 Non-Financial Public Sector |
| 1.1.1 Government | 2.1. Central Government | 3.1.1 Central Government |
| 1.1.2 Rest of General Government | 2.1.2 Rest of General Government | 3.1.2 Rest of General Government |
| 1.1.3 Public Non-Financial  Enterprises | 2.1.3 Public Non-Financial  Enterprises | 3.1.3 Public Non-Financial  Enterprises |
| 1.2 Financial Public Sector | 2.2 Financial Public Sector | 3.2 Financial Public Sector |
| 1.3 Central Reserve Bank | 2.3 Central Reserve Bank |  |

**Table S4: Summary Statistics – Average Monthly Aggregate Fiscal Variables by Country**

| country | revenue | std | Newly raised debt | std | expenditure | std |
| --- | --- | --- | --- | --- | --- | --- |
| Anguilla | **15.2** | *4.1* | **1.1** | *1.9* | **14.7** | *3.5* |
| Antigua & Barbuda | **60.6** | *24.0* | **6.0** | *27.1* | **66.7** | *25.8* |
| Belize | **70.1** | *28.6* | **3.8** | *4.7* | **63.6** | *28.5* |
| Barbados | **288.7** | *49.1* | **1.2** | *3.9* | **303.4** | *62.1* |
| Dominica | **33.6** | *13.4* | **3.4** | *4.7* | **29.0** | *4.5* |
| Grenada | **42.7** | *16.7* | **6.8** | *6.0* | **39.0** | *14.4* |
| Guatemala | **1163.4** | *291.2* | **11.6** | *10.2* | **978.9** | *330.6* |
| Guyana | **153.9** | *44.3* | **12.7** | *12.0* | **142.9** | *50.8* |
| St. Kitts & Nevis | **55.2** | *16.5* | **5.2** | *6.4* | **49.7** | *8.9* |
| St. Lucia | **69.0** | *10.9* | **2.6** | *2.5* | **61.5** | *11.6* |
| Montserrat | **3.8** | *0.7* | **8.5** | *5.6* | **9.2** | *2.4* |
| Nicaragua | **371.9** | *43.9* | **21.4** | *11.1* | **351.1** | *41.6* |
| El Salvador | **700.7** | *210.7* | **18.7** | *15.5* | **653.9** | *176.3* |
| Suriname | **187.7** | *101.6* | **6.5** | *16.0* | **196.5** | *95.0* |
| Trinidad & Tobago | **1133.6** | *520.7* | **N/A** | *N/A* | **1041.1** | *462.0* |
| St. Vincent & Grenadines | **41.6** | *8.6* | **2.3** | *3.1* | **39.6** | *7.6* |

Notes: (a) Mean in bold and standard deviation in italic font; (b) Figures in millions of (2010) $US

**Table S5: Summary Statistics – Hurricane Event Loss Data (millions of $US) and Storm Numbers; 2008-2018**

| event_name | month | year | country | damage | payout_total |
| --- | --- | --- | --- | --- | --- |
| Ike | September | 2008 | Turks and Caicos Islands | N/A | 6.3 |
| Earl | August | 2010 | Anguilla | 5.2 | 4.3 |
| Tomas | October | 2010 | Barbados | 0.7 | 8.6 |
| Tomas | October | 2010 | Saint Lucia | 4.3 | 3.2 |
| Tomas | October | 2010 | St Vincent & the Grenadines | 1.8 | 1.1 |
| Gonzalo | October | 2014 | Anguilla | 0.6 | 0.5 |
| Earl | August | 2016 | Belize | 12.9 | 0.3 |
| Matthew | September | 2016 | Barbados | N/A | 1.7 |
| Matthew | October | 2016 | Haiti | 29.8 | 23.4 |
| Matthew | September | 2016 | Saint Lucia | N/A | 3.8 |
| Matthew | September | 2016 | St. Vincent & the Grenadines | N/A | 0.3 |
| Otto | November | 2016 | Nicaragua | 0.8 | 1.1 |
| Irma | September | 2017 | Anguilla | 104.1 | 6.7 |
| Irma | September | 2017 | Antigua & Barbuda | 88.1 | 6.8 |
| Irma | September | 2017 | Bahamas | 0.0 | 0.2 |
| Irma | September | 2017 | Haiti | 0.0 | 0.2 |
| Irma | September | 2017 | St. Kitts and Nevis | 65.4 | 2.3 |
| Irma | September | 2017 | The Bahamas | 0.0 | 0.2 |
| Irma | September | 2017 | Turks & Caicos Islands | N/A | 14.9 |
| Maria | September | 2017 | Anguilla | 104.1 | 0.0 |
| Maria | September | 2017 | Antigua & Barbuda | 88.1 | 0.0 |
| Maria | September | 2017 | Barbados | 0.0 | 1.9 |
| Maria | September | 2017 | Dominica | 280.9 | 20.3 |
| Maria | September | 2017 | Saint Lucia | 0.5 | 0.8 |
| Maria | September | 2017 | St. Kitts & Nevis | 65.4 | 0.0 |
| Maria | September | 2017 | St. Vincent & the Grenadines | N/A | 0.2 |
| Maria | September | 2017 | Turks & Caicos islands | N/A | 0.4 |

Notes: (a) Figures in millions of (2010) $US; (b) Losses are estimated value at risk from direct damages.
